# Artificial Intelligence Should Not Prescribe Alone: A Risk-Stratified Pharmacy Governance Framework for Agentic Clinical Systems

**DOI:** 10.7759/cureus.115230

**Published:** 2026-08-26

**Authors:** Esteban Zavaleta-Monestel, José A Castro-Gamboa, Jeaustin Mora-Jiménez

**Affiliations:** 1 Research Department, Hospital Clínica Bíblica, San José, CRI; 2 Pharmacy Department, Hospital Clínica Bíblica, San José, CRI

**Keywords:** agentic artificial intelligence, artificial intelligence governance, clinical decision support, medication safety, pharmacy practice, prescribing

## Abstract

Agentic clinical artificial intelligence (AI) is moving beyond conversational assistance towards systems capable of generating medication-related recommendations and structured clinical actions. Although recent models have shown strong performance in simulated disease-management and medication-reasoning tasks, benchmark performance does not establish the safety of autonomous prescribing. Medication use depends on patient-specific information, local formularies, medicine availability, monitoring capacity, clinical workflows, and professional accountability. This editorial argues that medication-related AI should be governed according to clinical risk. Lower-risk functions may support medication reconciliation, monitoring and patient communication, whereas treatment initiation, dose adjustment, antimicrobial selection, therapeutic substitution and deprescribing should require explicit pharmacist and prescriber validation. Local prospective evaluation, transparent documentation, version control, incident monitoring, and sufficient professional authority are essential. Agentic AI may offer meaningful clinical support in medication-related workflows, but its role in prescribing should remain pharmacist-governed, locally validated, and auditable rather than autonomous.

## Editorial

Recent studies of medical artificial intelligence (AI) agents have moved the debate beyond conversational assistance towards systems capable of influencing longitudinal management and executing structured clinical actions [1,2]. These developments build on earlier work showing that large language models can conduct diagnostic dialogue and encode clinical knowledge [3,4]. The more urgent issue is whether these systems should influence medication selection, prescribing, dose adjustment, monitoring and deprescribing. Current evidence may support the supervised use of these systems in selected workflows, but it does not justify autonomous prescribing or independent medication-related decision-making [5]. Pharmacists may therefore be expected not only to verify AI-generated medication recommendations but also to govern systems evaluated in a limited range of structured, well-resourced clinical settings [1,2]. Agentic clinical AI should therefore be treated as a high-risk intervention within the medication-use process. Its implementation should require pharmacist oversight, local validation, traceability and clearly defined accountability [6,7].

Medication use is not a simple information-retrieval task. It is a continuous clinical process involving diagnosis, therapeutic selection, prescribing, preparation, dispensing, administration, monitoring, adherence assessment, treatment evaluation, modification, deprescribing and communication with patients and caregivers. Each decision must be interpreted within the patient’s clinical condition and the realities of the health system, including renal and hepatic function, comorbidities, allergy history, drug interactions, local formularies, institutional protocols, availability of medicines, antimicrobial resistance patterns and patient preferences. A medication order may be guideline-concordant and pharmacologically plausible yet still be unsafe, unavailable, unaffordable, duplicated, incorrectly timed or impossible to administer. These contextual judgements are central to pharmacy practice and explain why medication-related AI should be treated as a high-risk intervention rather than as a natural extension of digital convenience.

Articulate Medical Intelligence Explorer (AMIE) and Medical Intelligence for Reasoning and Action (MIRA) illustrate how rapidly agentic AI is approaching medication-related decision-making. AMIE extended conversational AI beyond diagnostic dialogue to longitudinal disease management and medication reasoning, whereas MIRA operated within a sandboxed electronic health record environment in which it could request investigations, interpret results, prescribe medicines, recommend procedures, and make admission or discharge decisions [1,2]. Their significance for pharmacy practice lies not only in what these systems can now do, but also in the safeguards required when AI-generated recommendations begin to influence executable medication-related actions.

In simulated multi-visit management scenarios, AMIE demonstrated strong performance in treatment planning and medication reasoning. Its evaluation included RxQA, a benchmark derived from national formularies in the United States and the United Kingdom and validated by board-certified pharmacists; AMIE outperformed primary care physicians on higher-difficulty questions [2]. These findings suggest that conversational AI may become useful for structured therapeutic support, but they do not establish safe prescribing in routine practice. Answering a formulary-based question or producing a guideline-aligned recommendation is fundamentally different from managing a patient with multimorbidity, incomplete documentation, changing organ function, local restrictions, medicine shortages and evolving clinical priorities.

MIRA presents a more consequential transition because it moves from advice to structured actions within an electronic health record-like environment [1]. Once an AI system can draft or initiate medication orders, safety depends not only on whether its reasoning is clinically plausible, but also on whether each action is complete, executable, traceable, locally appropriate and reviewed before reaching the patient. MIRA’s medication safety results were encouraging but not definitive. The evaluation remained confined to a sandbox, simulated cases, predefined safety domains and a subset of outputs. It identified three instances classified as therapeutic duplication, although each was judged clinically reasonable; route specification was the least accurate prescription field [1]. These apparently small weaknesses may become clinically important when repeated at scale or combined with workload pressure, incomplete records and automation bias.

Transferability is a central limitation of the current evidence. AMIE’s medication-reasoning benchmark drew on national formularies from the United States and the United Kingdom, whereas MIRA was evaluated using retrospective clinical records within a structured electronic health record sandbox [1,2]. These designs are appropriate for early technical evaluation, but they do not establish performance across health systems with different formularies, prescribing rules, patient populations, documentation practices, staffing models, procurement constraints or levels of digital maturity. An AI-generated recommendation may therefore be clinically coherent yet locally inappropriate if a medicine is unavailable, an institutional protocol differs, essential monitoring cannot be obtained, relevant information is absent from the record or the proposed action cannot be integrated safely into the existing workflow. Performance demonstrated elsewhere should be interpreted as evidence of technical capability, not as proof of local clinical safety; deployment should be preceded by evaluation against local formularies, protocols, documentation quality, patient populations, workflows and pharmacy capacity [6,7].

The presence of a human reviewer should not be treated as sufficient evidence that an AI-enabled prescribing workflow is safe. ‘Human-in-the-loop’ may describe anything from rapid acknowledgement of an alert to an independent clinical assessment with authority to modify or reject the recommendation. Effective oversight requires sufficient time, access to relevant information, appropriate training, clear escalation pathways and institutional support for professionals who override the system. Without these conditions, human review risks becoming a procedural formality rather than a meaningful safety barrier [6,7].

This distinction is particularly important for pharmacy services. If pharmacists must verify an increasing number of AI-generated recommendations while maintaining dispensing, clinical, stewardship and operational responsibilities, the technology may add cognitive and documentation work rather than reduce it. A process that is feasible in a well-staffed department with informatics support may be unsafe or impracticable where staffing is limited, records are fragmented, or no protected time is available for clinical validation. Institutions should assess this workload before implementation and provide staffing, training and decision-making authority proportionate to the risk of the task.

Automation bias may further weaken human oversight, particularly when AI outputs appear precise, structured and guideline-based. Under time pressure, clinicians may accept recommendations without fully examining their assumptions, missing data or local applicability. Pharmacists must therefore retain the authority to interrogate and reject AI-generated recommendations [7].

Medication-related AI is only as reliable as the clinical and operational information available to it. Medication histories may be incomplete, allergies poorly characterized, laboratory results delayed and non-prescription or complementary medicines absent from the record. Formularies, institutional protocols, medicine availability and antimicrobial resistance patterns may also change more rapidly than the information embedded in a system. When missing or outdated data are treated as complete and current, a recommendation may appear internally coherent while being unsafe or impossible to implement [7].

Before a recommendation is acted upon, the system should disclose the patient data, assumptions, clinical guidance and local operational information on which it relied. It should identify missing variables that could materially alter the recommendation, including renal or hepatic function, pregnancy status, recent laboratory results, previous treatment response, allergy details, concomitant medicines or local availability. When critical information is unavailable, the appropriate output may be a request for clarification or further assessment rather than a treatment recommendation.

Building on these evidence-based concerns, we propose a risk-stratified governance framework for medication-related AI. This framework is intended to operationalize existing safety and governance principles and should be understood as an author-proposed model rather than a prospectively validated clinical standard.

Within this proposed framework, the required level of human review should increase with the potential severity, irreversibility and monitoring complexity of the proposed action. Decisions involving antimicrobials, anticoagulants, insulin, opioids, chemotherapy, immunosuppressants, pediatric dosing, renal replacement therapy, pregnancy and lactation or deprescribing in frail older adults may cause substantial harm when based on incomplete information or implemented without timely monitoring. AI systems should not independently initiate, discontinue or substantially modify these therapies. Health systems should classify medication-related AI functions by clinical risk and require pharmacist and prescriber review, with stricter controls for therapies involving narrow therapeutic ranges, complex dose individualization or rapid detection of toxicity.

Medication-related AI also creates documentation and accountability challenges. When a recommendation is accepted, modified or rejected, the final decision and the professional responsible for it should be recorded, clearly distinguishing the system’s original output from the action ultimately authorized. AI-generated recommendations should be identifiable, time-stamped and linked to the patient information available when they were generated. This traceability is necessary for investigating harm and detecting recurring failures [6,7].

Because AI-enabled systems may change over time, institutions should also document the deployed version and evaluate the effects of updates. The FDA’s predetermined change control plan framework provides one regulatory example of prospective documentation and validation of planned modifications, although it applies specifically to AI-enabled device software functions [8].

Accountability should remain with the health system and the professionals who authorize medication-related actions, but responsibility cannot be transferred to individual pharmacists without corresponding authority over system selection, implementation, restriction and suspension. Pharmacists overseeing AI-generated decisions should have access to performance data, incident reports and escalation mechanisms, including the authority to restrict or halt a function when safety concerns emerge. Governance must define who reviews the system, approves its clinical use, responds to failures and can withdraw it from clinical practice [6,7,9].

Prospective validation is essential before medication-related AI is introduced into routine prescribing workflows. Simulated cases, retrospective analyses and benchmarks can establish technical capability, but they cannot determine whether a system reduces harm under real clinical conditions [1,2,5]. Evaluation should occur within the workflows, patient populations, formularies, staffing models and information systems in which the technology will be used [6,7,9]. Outcomes should extend beyond agreement with guidelines or clinicians to include intercepted prescribing errors, preventable adverse drug events, monitoring failures, clinically inappropriate recommendations, professional override rates, alert burden, pharmacist workload, treatment delays and patient outcomes. Performance should also be examined across patient groups, clinical services and levels of documentation completeness rather than reported only as an overall average.

**Figure 1:**
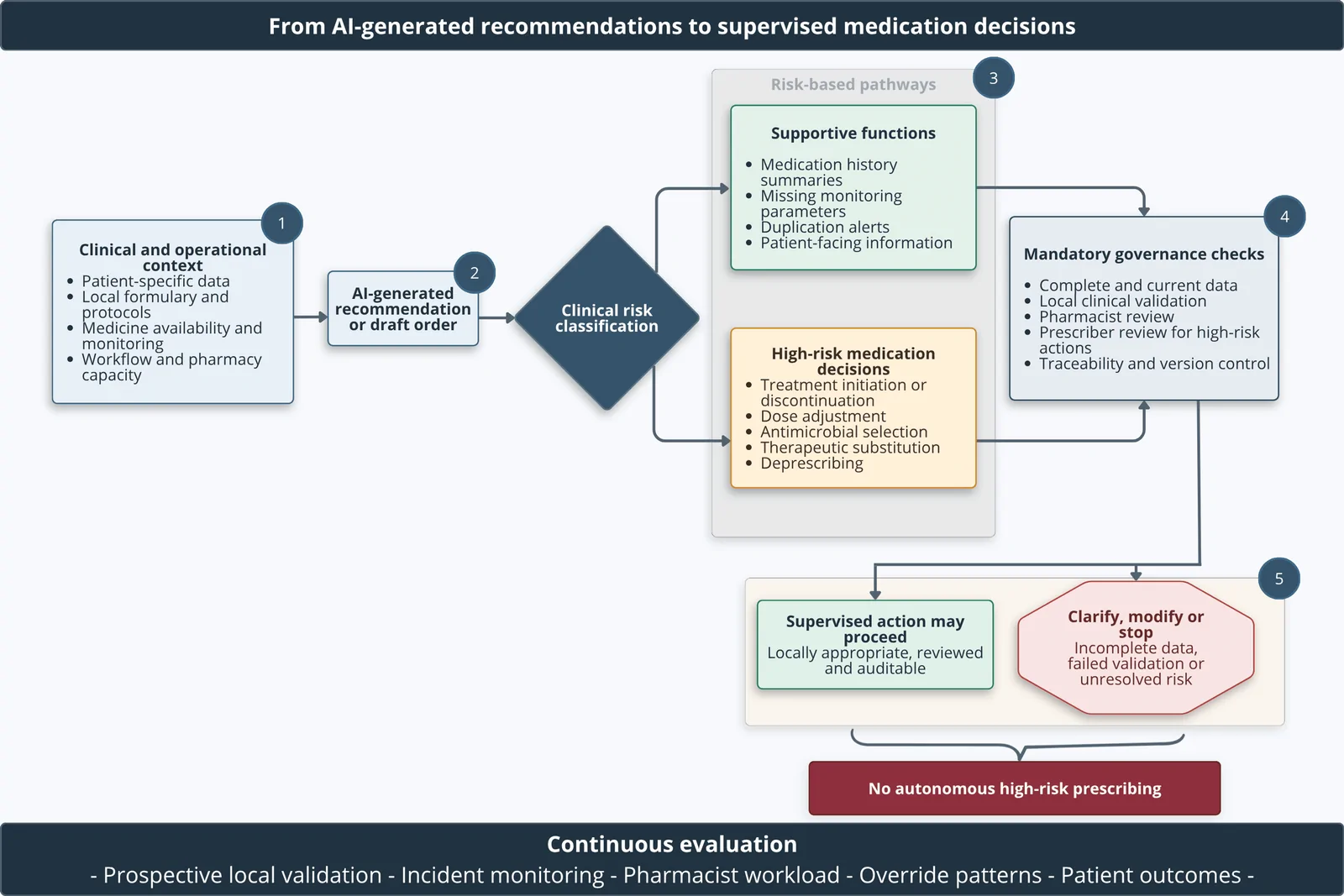
Risk-stratified pharmacy governance framework for medication-related agentic artificial intelligence (AI). Clinical and operational context informs an AI-generated recommendation or draft order, which is subsequently classified according to clinical risk. Supportive and high-risk medication functions undergo mandatory governance checks, including assessment of data completeness, local clinical validation, pharmacist review, prescriber review for high-risk actions, traceability and version control. Actions may proceed only after appropriate professional review, whereas incomplete data, failed validation or unresolved risk should prompt clarification, modification or cessation. Fully autonomous high-risk prescribing remains outside the proposed framework. Continuous evaluation feeds back into the clinical and operational context through local validation, incident monitoring, workload assessment, override analysis and patient outcomes. The figure was manually designed by the authors using Canva (Canva Pty Ltd., Sydney, Australia).

Implementation should proceed according to the clinical risk of the AI function. Lower-risk applications may include summarizing medication histories, identifying missing monitoring parameters, detecting possible duplication, drafting patient-facing information or generating questions for professional review. Functions involving treatment initiation, dose adjustment, antimicrobial selection, therapeutic substitution or deprescribing require stronger evidence and mandatory pharmacist and prescriber validation [9]. Within this risk-based approach, the WHO Medication Without Harm challenge provides a relevant medication-safety rationale for prioritizing high-risk situations [10]. Figure 1 summarizes the proposed risk-stratified governance pathway for medication-related agentic AI.

Pharmacy practice should therefore move from passive verification of individual outputs to active governance of medication-related AI [9]. This does not require a specialized AI committee in every institution; existing pharmacy and therapeutics committees, medication-safety programmes, antimicrobial stewardship teams, clinical governance structures and digital health committees can establish review requirements, monitor incidents and restrict unsafe functions. Governance should reflect local resources without lowering safety standards or bypassing local evaluation.

Professional education must develop in parallel. Pharmacists need the ability to evaluate AI-generated recommendations, recognize automation bias, identify missing or unreliable data, assess local applicability and distinguish technical performance from evidence of clinical safety. They need not become software engineers, but they should understand a system’s intended use, validation population, limitations, update processes and audit mechanisms [9].

Agentic AI may improve medication reconciliation, monitoring, formulary navigation, patient communication and therapeutic decision support. These potential benefits should not be confused with authority to prescribe independently. Medication decisions remain embedded in clinical uncertainty, local availability, patient preferences and professional accountability; accordingly, agentic AI should remain a supervised, locally validated and auditable clinical tool rather than an independent medication decision-maker.
